# Foods and Nutrients at Risk for Insufficient Intake by Community-Dwelling Healthy Older Women Eating Alone and Together in Japan—A Preliminary Finding

**DOI:** 10.3390/nu15102391

**Published:** 2023-05-19

**Authors:** Tomoya Takiguchi, Muneko Nishijo, Noriko Kaneko, Katsushi Yoshita, Yusuke Arai, Noboru Demura, Yoshikazu Nishino

**Affiliations:** 1 Department of Public Health, Kanazawa Medical University, Uchinada 920-0293, Japan; ttakiguc@kanazawa-med.ac.jp (T.T.); ynishino@kanazawa-med.ac.jp (Y.N.); 2 Faculty of Nursing, Ishikawa Prefectural Nursing University, Kahoku 929-1210, Japan; kaneko@ishikawa-nu.ac.jp; 3 Department of Public Health, Nutrition, School of Human Life and Ecology, Osaka Metropolitan University, Osaka 558-8585, Japan; yoshita@omu.ac.jp; 4 Department of Nutrition, Chiba Prefectural University of Health Sciences, Chiba 260-0801, Japan; yusuke.arai@cpuhs.ac.jp; 5 Department of Oral and Maxillofacial Surgery, Kanazawa Medical University, Uchinada 920-0293, Japan; n-demura@kanazawa-med.ac.jp

**Keywords:** eating alone, dental status, nutrient intake, food consumption, older adults

## Abstract

Eating alone and poor dental status have been reported to influence dietary intake in older adults. First, we compared nutrient and food intake and dental markers between women eating alone and together, who participated in a home health management program conducted by Kanazawa Medical University. The results showed the significantly higher intake of fresh fruit and some micro-nutrients and a lower decayed, missing, and filled teeth (DMFT) index (better dental status) in women eating alone after adjusting for age, suggesting that dental status may mediate the association between commensality and dietary intake. Then, we investigated nutrients and foods at risk for insufficient intake and associated with increasing dental markers. The risks for the insufficient intake of protein and *n*-3 and *n*-6 polyunsaturated fatty acids (PUFAs) were significantly increased with an increasing DMFT index. The risk for *n*-3 PUFA intake also increased with increasing numbers of missing teeth in women. Foods at risk for insufficient consumption included beans for women with an increasing DMFT index and green and yellow vegetables, fresh fruits, and meat and fish for women with increasing numbers of missing teeth. These findings suggest that good health management, including the treatment of decayed teeth, is important for the prevention of malnutrition in community-dwelling healthy older women.

## 1. Introduction

Older adults are at risk for malnutrition because of physiological and metabolic changes that progress during aging, particularly those that occur in the digestive organs, such as decreased gastric acid secretion and salivary production, which lead to the insufficient digestion and absorption of nutrients, and appetite loss due to a diminished sense of smell and taste [1]. In a population-based study in Germany of more than 1000 community-dwelling older adults aged ≥65 years, frequent subclinical micronutrient deficiencies, such as the lower intake of iron, folate, vitamin D, and vitamin B12, were reported, although malnutrition indicated by a low body mass index (BMI) due to insufficient protein and energy intake (macronutrient deficiency) was not common [2]. In the same study, physical inactivity and frailty were indicated as relevant factors associated with subclinical microdeficiencies in this older German population [2].

In 2021, Bjorwall et al. reviewed the literature and found that eating alone and eating together (commensality) were key factors related to eating behavior that could lead to malnutrition in community-dwelling older adults [3]. In older Japanese adults aged ≥65 years, Tani et al. reported in 2015 that men eating alone were more likely to be underweight (BMI < 18.5) than men eating together, although no clear association was found between eating alone and the prevalence of underweight in women [4]. These findings suggest macronutrient deficiency only in men. However, the authors of this Japanese study suggested that micronutrient deficiency might be possible in women who eat alone as a result of more frequent unhealthy dietary habits, such as skipping meals, in comparison with women eating together [4].

The alteration of oral health status with aging, such as the increased prevalence of caries, periodontal disease, and edentulism, and consequent decreased chewing ability due to the increased loss of teeth directly affects nutrition in older adults [5,6]. In this regard, many studies that investigate associations between dental status and dietary intake have been conducted in the United Kingdom (UK), the United States, and Asian countries including Japan. Dental parameters including edentate/dentate, the numbers of present teeth or missing teeth [7,8,9,10], self-reported chewing ability [11,12], and observational chewing ability [13,14] have been reported to be associated with lower dietary intakes in community-dwelling older adults. Particularly in Japanese studies, chewing ability evaluated by color-changeable chewing gum [13] or small numbers of posterior occluding pairs of teeth [14] have been investigated in relation to lower intakes of various kinds of foods and nutrients, including minerals and vitamins, in older adults in comparison with their counterparts with normal oral health status.

Among older adults aged ≥75 years who participated in the Adult Dental Health Survey 2009 in the UK, older adults who lived alone more frequently wore dentures, self-reported a poor oral status, and had poor dental care attendance in comparison with their co-habiting counterparts [15]. In Sweden, a Swedish National Quality Register Study to investigate relationships between quality of life and oral health among older adults aged ≥65 years reported that living alone was one of the relevant factors in having a higher risk of oral health problems [16]. These results suggest that older adults living alone and, ipso facto, eating alone may have an insufficient intake of foods and nutrients, and that this may be related to oral health problems.

Against this background, we first compared food and nutrient intake and dental markers among Japanese older women living and eating alone and together and found different dietary and dental statuses between them, suggesting an association between commensality (eating alone and together) and dental status. Because the subjects enrolled were participants in a home health management program organized by our department at Kanazawa Medical University, the number of men who ate alone was too small for statistical analysis, with only one man. We then investigated the risks associated with dental parameters for insufficient food and nutrient intake in women under the hypothesis that dental status may mediate the association between commensality and dietary intake. At this time, the reference levels of nutrients recommended from the standpoint of good health in healthy older adults by the Japanese government [17] were used for defining cut-off values of insufficient intake.

## 2. Materials and Methods

### 2.1. Study Subjects

**Figure 1:**
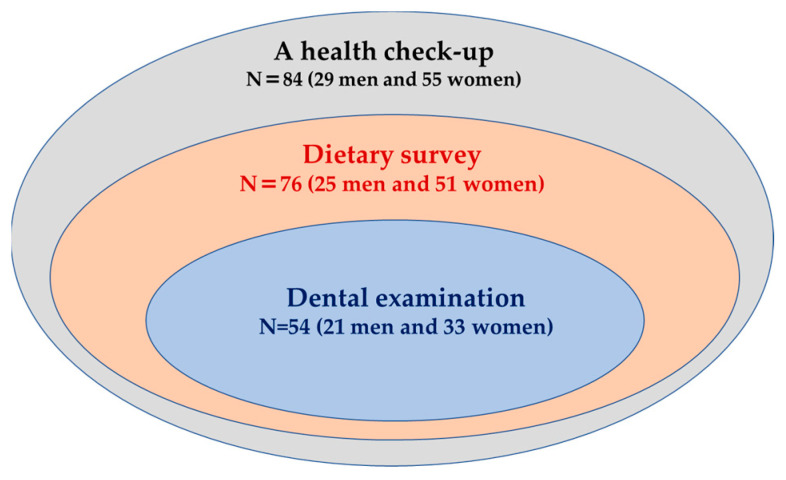
Participants of the health impact survey consisting of a health check-up, dietary survey, and dental examination.

A total of 84 independent older adults (29 men and 55 women) aged 65 years and older who resided in Uchinada town, Japan, and participated in home health management conducted by the Department of Public Health and Epidemiology, Kanazawa Medical University, were invited to participate the health impact survey consisting of a health check-up, dietary survey, and dental examination in May 2019. All of them underwent a health check-up including body size measurement, blood and urinary tests, blood pressure measurement, electrocardiogram, and chest X-ray examination (Figure 1). The number of participants of the dietary survey after the health check-up were 76 older adults (25 men and 51 women) who had completed dietary records at home (Figure 1).

A total of 54 older adults (21 men and 33 women) from participants to the dietary survey underwent an oral examination (Figure 1). The participant rate of the dental survey was lower, particularly in women (60%), compared to that of the dietary survey because women who visited dentists for regular dental check-up did not want to be examined again in the current survey.

The information on living alone or together with family, smoking habits, and educational history were obtained from records of home health management. In the dietary survey, the information on eating alone or together was collected by dietitians.

The study design and all procedures were approved by the Medical Research Ethics Review Committee of Kanazawa Medical University (No. I522, 24 June 2020). Written informed consent was obtained from all subjects.

### 2.2. Measurements

#### 2.2.1. Dental Examination

An intra-oral examination was conducted by a single dentist using a dental mirror and a dental probe. Participants’ teeth were recorded as sound, decayed, missing, and filled according to the diagnostic criteria of the Japanese national survey titles “Survey of Dental Diseases” [18]. Third molars, extractions for orthodontic treatment, and areas considered innate defects were excluded from missing teeth. The DMFT index numerically expresses the prevalence of dental caries and is calculated from the sum of numbers of decayed teeth, missing teeth, and filled teeth. A higher value of DMFT index indicates poorer dental status [19,20].

#### 2.2.2. Dietary Survey

Subjects were required to record all foods and beverages including ingredients and portion sizes for 2 days. At the site of the survey, dietitians interviewed the subjects to confirm their records and the amount of each ingredient, using standard food models (Iwai Sample, Osaka, Japan) to improve accuracy and estimate their dietary intake. Total energy intake, macronutrient intake and energy ratios (balances), and micronutrients including minerals and fatty acids were estimated by the nutritional calculation software “Shokuji-Shirabe 2011”, developed by the National Institute of Health and Nutrition. At this time, intakes were calculated after accounting for changes in weight and nutrient content arising from cooking. To define the lower limit of recommended intakes, we used the Dietary Reference Intakes for Japanese (2015) [17] for each nutrient. The cutoff values for beans, fruit, and vegetables were set according to intake levels recommended in the national health promotion named Healthy Japan 21 [21]. For other foods, such as meat and fish, intakes were set by referring to the recommended intakes (for 1600 calories per day) in the handbook based on the 4-food-groups scoring method [22].

### 2.3. Statistical Analysis

STATA/MP 16.1 (Stata, College Station, TX, USA) was used for statistical analysis. Adjusted comparisons of demographic factors, dental parameters, and nutrient and food intakes were compared between older adults eating alone and together after adjusting for age using general linear model (analysis of covariance with age as a covariate) in women alone because of the small number of men eating alone. A logistic regression model was used to analyze the risks (odds ratios) for insufficient nutrient and food intakes associated with increasing dental parameters after adjusting for age in women alone because recommended values for nutrients were different between men and women [17]. In the current analysis, smoking status and educational history, which are well-known confounding factors in dental and nutrition status, were not adjusted at this time because there were very few smokers (two men and no women) and no significant correlations between dental markers or nutrients and educational history in our subjects. The significance level was set at 5% (*p* < 0.05).

## 3. Results

One man (4.2%) and nineteen women (37.3%) were living alone. All older adults living alone prepared food by themselves and ate alone on almost all days except for a few days per month when meeting family members or friends living in different areas. Almost all older adults who lived with others (95.8% men and 90.6% women) were eating with their spouse, except for one man and two women who lived and ate with their children and one woman who lived and ate with her sister. Taken together, in the current study, all subjects living alone ate alone, and all subjects living together ate together. In the results that follow, “eating alone or together” means “living and eating alone or together”.

### 3.1. Comparisons of Body Size and Food and Nutrient Intake between Older Adults Eating Alone and Together in Each Sex

According to sex and commensality (eating alone or together), the means with standard deviations (SD) of age and BMI and the frequencies (%) of underweight (BMI < 18.5) and overweight (BMI ≥ 25.0), as defined by the Japan Society for the Study of Obesity, are shown in Table 1.

Only one man ate alone; therefore, no comparison could be made between men eating alone and those eating together. In women, no significant differences in age, body size, or the frequency of cases with low and high bodyweight were found between older adults eating alone and together. Daily food intake (g) in each food group was also compared between women living and eating alone and together (Table 1). However, only fresh fruit intake was significantly higher in women eating alone, even after adjusting for age (*p* < 0.05), in comparison with women eating together.

No significant difference in macronutrient intake was found between women eating alone and together (Table 1). With regard to micronutrients, however, the intakes of potassium, magnesium, Vitamin K, folate, and total dietary fiber were significantly lower in women eating together after adjusting for age in comparison with women eating alone (Table 1).

### 3.2. Comparisons of Body Size and Food and Nutrient Intake between Men and Women Eating Together

To estimate the gender differences of nutritional status, body size and food and nutrient intake were compared between men and women eating together (Table 1; statistical test results not shown in the table).

The prevalence of overweight (BMI ≥ 25) and mean rice intake were significantly higher (*p* = 0.025 and *p* = 0.006, respectively) in men eating together compared with women after adjusting for age. Additionally, men eating together showed significantly higher energy (*p* = 0.001), carbonate (*p* = 0.001), and carbonate balance (*p* = 0.005) and significantly lower protein balance (*p* = 0.008) and fat balance (*p* = 0.028) compared with women eating together. These results indicate that higher energy intake due to higher rice intake might be related to a high prevalence of overweight in men eating together in this cohort.

### 3.3. Comparisons of BMI, Dental Parameters, and Food and Nutrient Intake between Female Dental Examination Participants Eating Alone and Together

Because of the smaller number of participants in the dental examination, including only one male participant who ate alone, the comparisons of health and nutritional markers including dental parameters between groups eating alone and together were, once again, only performed in women (Table 2).

Although there was no difference in BMI-related markers between the two groups, dental status, such as that defined by the DMFT index, and, particularly, the number of untreated, decayed teeth, was significantly lower in women eating alone after adjusting for age. Regarding food intake, women eating alone ingested significantly more fresh fruits and seaweed than women eating together. These results suggest that dental and nutritional status are better in women eating alone.

Regarding nutrient intake, women eating alone ingested significantly more sources of energy, protein, and fat compared with women eating together. Fat balance was significantly higher and carbonate balance significantly lower in women eating alone than in women eating together. Similarly, the intakes of vitamin A, E, K, and C; folate; *n*-3, *n*-6 polyunsaturated fatty acids (PUFAs); and total dietary fiber were significantly higher in women eating alone compared with women eating together after adjusting for age (Table 2).

### 3.4. Increased Risks for Insufficient Nutrient and Food Intake Associated with Dental Status in Women

Significant inverse associations between macronutrient parameters, such as protein and/or fat intakes and balance, and dental parameters were found in female participants of the dental examination, whereas there were no significant associations between them in all women. With regard to micronutrients, the intake levels of phosphorus, iron, zinc, *n*-3 and *n*-6 PUFAs, vitamin A, vitamin E, folic acid, and total dietary fiber were also significantly and inversely associated with both the DMFT index and/or the number of missing teeth.

Then, we analyzed the risks (odds ratios) for insufficient nutrient intake associated with increasing dental parameters using binary logistic analysis, and the results for women are shown in Table 3. In addition, a risk analysis for insufficient intake was performed only for protein because of the lack of reference values for good health from other macronutrients [17].

In women, after adjusting for age, the risk (odds ratio) of the insufficient intake of protein (<55 g/day) was 1.2 and significantly associated with an increasing DMFT index. The odds ratios for the insufficient intakes of *n*-3 PUFA (<1.9 g/day) and *n*-6 PUFA (<7 g/day) were 1.18 and 1.16, respectively, and significantly increased with an increasing DMFT index in women (Table 3). The number of missing teeth was also significantly associated with an increased odds ratio for the insufficient intake of *n*-3 PUFA, suggesting a 1.19 times higher risk with the increasing number of missing teeth in women (Table 3).

The numbers of decayed_teeth were significantly associated with a lower intake of Vitamin B12 with an increased odds ratio of 3.45 in women.

Regarding the risk analysis for insufficient food intake, a lower intake of beans (<100 g/day) was significantly associated with an increasing DMFT index, with an odds ratio of 1.14 for an increase in the DMFT index. The numbers of missing teeth were significantly associated with the lower intake of green and yellow vegetables (<120 g/day), fresh fruits (<150 g/day), and meat and seafood (<100 g/day), with increased odds ratios of 1.17, 1.25, and 1.35, respectively (Table 3).

## 4. Discussion

### 4.1. Commensality (Eating Alone or Together) and Food and Nutrient Intake

No significant difference in macronutrient intake or the frequency of underweight and overweight was found between women eating alone and together. However, fresh fruit consumption and the intakes of folate, total dietary fiber, and several micronutrients, including potassium, magnesium, and Vitamin K, were significantly higher in women eating alone compared to women eating together. In addition, the risks (odds ratios) for insufficient fresh fruit intake (<150 g/day) and folate intake (<240 g/day) in women eating alone were significantly lower than in women eating together. These results suggest better nutrient intake in older women eating alone than in those eating together, which is inconsistent with the study results of increased malnutrition risks associated with eating alone in previous reports involving older adults [3,4].

One probable reason for these unexpected results is that women in the current study have participated in our home health management program, including the nutrition survey, and practice good dietary behavior. In particular, women living and eating alone are taking care of themselves regarding their diet because they have come to recognize that they should remain healthy enough to stay at home independently. In contrast, women eating together prepare food primarily for their families and consider others’ tastes and health statuses. Almost all of them were living with their husbands, whose micronutrient intakes were similar to theirs, although husbands consumed more carbohydrates due to higher rice intake.

More specifically, the mean macronutrient intake in women eating alone who participated in the dental examination was higher than that in all women eating alone (Table 2), suggesting that women who saw an opportunity to improve their health status and eat nutritional foods may have agreed to participate in the dental examination.

Dental status as a health indicator was also better in women eating alone than in those eating together because of the significantly lower number of untreated, decayed teeth. These results also support the notion that the women living alone in the current study were more inclined toward self-care, including dental care attendance, than women living together.

In the UK, it was previously reported that men living and eating together who were responsible for preparing food but had poorer cooking skills had lower energy intake and specific nutrients [23]. However, in the current study, we were unable to analyze the association between commensality and nutritional status in men because only one male participant was living and eating alone. Future recruitment should be extended to more men living and eating alone among the older adult population, and their food consumption and nutrient intake should be investigated using methods that are easier than the “dietary diary” approach, such as taking photos of meals.

### 4.2. Associations between Dietary Intake and Dental Parameters

#### 4.2.1. Nutrient Intake and Dental Parameters

In women, the intake of various kinds of macro- and micronutrients, including protein, trace elements, *n*-3 and *n*-6 PUFAs, and vitamins, significantly decreased with an increasing DMFT index and increasing numbers of missing teeth. The risk for insufficient intake of protein and *n*-3 and *n*-6 PUFAs increased significantly with an increasing DMFT index. Particularly for *n*-3 PUFA intake in women, this risk increased with increasing numbers of missing teeth. Regarding foods, the increased risk for insufficient meat and fish consumption was associated with increasing numbers of missing teeth, and the risk for the insufficient consumption of beans increased significantly with an increasing DMFT index, albeit only in women.

Consistently with these results, Yoshihara et al. performed a survey of nutritional intake using a precise weighing method for ≥74-year-old residents in another coastal area in Japan and reported that numbers of present teeth were significantly associated with various nutrients, including protein, minerals, and vitamins, among subjects of both sexes after adjusting for sex, smoking habit, and education [10]. They also reported that the ingestion of seafood and vegetables was significantly lower in subjects with <20 present teeth compared with their counterparts with ≥20 teeth. These results suggest that increasing numbers of missing teeth may influence intakes of vegetables and seafood, which contribute to sufficient protein intake in Japanese older adults.

Although no previous studies investigated the association between nutritional intake and the DMFT index in older adults, in the current study, women with a higher DMFT index had an increased risk for the insufficient intake of protein, *n*-6, and beans, suggesting that not only numbers of missing teeth but also numbers of decayed and filled teeth caused by dental caries may influence chewing ability and thus decrease nutritional intake in healthy older women.

#### 4.2.2. *n*-3 and *n*-6 PUFA Intake and Dental Parameters

Iwasaki et al. followed up adults aged 74 years and older who participated in the survey performed by Yoshihara et al. [10] and reported that subjects with a lower intake of docosahexaenoic acid, one of the *n*-3 PUFAs, developed progressive periodontal disease because of the aggravated inflammation of the oral environment during a follow-up period of 5 years [24]. Iwasaki et al. also showed that *n*-3 PUFA intake from fish was significantly lower among ≥80-year-olds with ill-fitting dentures or compromised dentition compared to similarly aged people with good dentition [14]. However, among the present subjects, seafood intake was not insufficient, and *n*-3 PUFA intake was highly correlated with seafood intake. In general, oily fish, which contains *n*-3 PUFA, was not often eaten by older adults because they prefer to consume white-flesh fish with a lower fat content. Taken together, older women with poor dental status, even when living in a coastal area, should be advised to increase their intake of *n*-3 PUFA via fish for the prevention of not only cardiovascular diseases [25] but also oral inflammatory diseases such as periodontitis [24].

The insufficient intake of *n*-6 PUFA associated with dental parameters was also suggested to occur in women. Because of the high correlation of *n*-6 PUFA intake with the consumption of beans, which is a food at risk of insufficient consumption, *n*-6 PUFA intake may be insufficient in women with an increased DMTF index. In addition, insufficient protein intake was also significantly associated with a higher DMFT index. Therefore, the consumption of tofu and bean products should be recommended for older women with a high DMFT index to increase both protein and *n*-6 PUFA intake.

#### 4.2.3. Fruit and Vegetable Intake and Dental Parameters

The increased risk of lower fresh fruit intake associated with both dental markers was observed, although our subjects had no increased risk of lower vitamin C intake because there was no significant increased risk of lower total vegetable intake, which is another source of vitamin C. The lower intakes of fruits, vegetables, vitamin C, and potassium have been reported in Korea, where older adults self-reported chewing difficulties [12]. These authors also compared their percentages of nutrient intakes, relative to the recommended levels of dietary reference intakes, for Koreans between groups with and without chewing difficulty, and they reported lower intakes of total energy and nutrients other than vitamin C and potassium, including calcium, which was inconsistent with our results that showed an increased risk for the lower intake of only limited nutrients. The difference between our results and those of Kwon et al. may be related to differences in dental status, which was worse in their subjects who reported chewing difficulty [12].

In the United States, people wearing dentures were reported to eat less fruit compared with dentate individuals, although psychosocial factors such as attitude and self-identity contributed more than chewing ability to this increased fruit intake [26]. In Japan, self-efficacy for designing meals and the awareness of recommendations for fruit and vegetable intake were reported to be important for the entire aged population [27]. In the present study, however, dental status including dental caries may be an important factor in fruit intake among women who have had the opportunity to improve their lifestyle using the home health management program.

The risk of low intakes of green and yellow vegetables was significantly associated only with women with a high DMFT index and a significantly lower intake of vitamin A and vitamin E, which are ubiquitous in green and yellow vegetables. In the total cohort, there were significantly increased odds ratios for the insufficient intake of green and yellow vegetables alongside an increase in both renal markers (DNFT index > number of missing teeth) and an inverse correlation between dental markers and vitamin E intake, suggesting that poor dental status in both sexes may decrease vitamin E because of the lower consumption of green and yellow vegetables.

### 4.3. Strengths and Limitations

The main strength of this study is the dietary survey method; diet records, which are not easy for older adults, were successfully achieved. Additionally, dietitians who interviewed the current cohort have supported our home health management program for more than 10 years and know their eating habits and common diet well enough to improve the accuracy of their records and estimate their dietary intake. With these records, we could perform a risk analysis of the insufficient intake of these micronutrients for which reference levels are established for each sex. Another strength is our cohort of targeted subjects comprising older healthy adults with an interest in health management, including oral health. In the present study, we showed that key micronutrients and foods were at risk of insufficient intake even in older adults living a healthy lifestyle.

Despite these strengths, the small sample size, particularly for men, is a limitation of the study because it decreases statistical power, especially in an analysis with multiple covariates. Indeed, the number of men eating alone amounted to only a single individual. Even for women, the sample size was too small for statistical analysis using the model with two main factors, the commensality factor and dental factor. In future surveys, we will enroll larger numbers of older adults to include more men living and eating alone.

## 5. Conclusions

In older women living a healthy lifestyle with good health management, poor commensality (i.e., living and eating alone) was not a risk factor for insufficient food consumption and nutrient intake. However, poor dental status, indicated by the DMFT index and the numbers of missing teeth, decreased the ingestion of beans, green and yellow vegetables, and fresh fruits and increased the risk for the insufficient intake of protein and *n*-3 and *n*-6 PUFAs. These findings suggest that good health management, including the treatment of decayed teeth, is important for the prevention of malnutrition in community-dwelling older adults.

## Figures and Tables

**Table 1:** Comparisons of body size and food and nutrient intake between older adults eating alone and together.

Category	Health and Nutrition Markers	Men				Women				*p*
		Alone (N = 1)		Together (N = 24)		Alone (N = 18)		Together (N = 33)		
		Mean, %	SD	Mean, %	SD	Mean, %	SD	Mean, %	SD	
Age and habits	Age (years)	82.0		77.1	4.6	77.7	4.9	75.3	5.8	
	Present smokers (%)	100.0		20.8		0.0		0.0		
Body size	Height (cm)	167.0		164.2	5.8	149.7	4.6	151.4	5.4	
	Weight (kg)	72.5		63.7	9.6	50.4	7.7	49.7	8.4	
	BMI	26.0		23.6	3.3	22.4	3.0	21.7	3.3	
	BMI < 18.5 (%)	0.0		8.3		5.6		9.1		
	BMI ≥ 25 (%)	100.0		41.7		16.7		12.1		
Food intake	Rice (g)	560.0		282.3	107.7	233.9	124.2	199.9	105.4	
	Beans (g)	10.0		51.3	38.9	75.6	69.4	58.1	52.7	
	Green vegetables (g)	217.5		129.5	90.0	157.1	75.4	133.9	83.6	
	Other vegetables (g)	24.0		122.6	71.9	151.2	90.6	137.4	73.0	
	Fresh fruits (g)	15.0		90.4	47.9	151.2	81.6	97.7	62.9	*
	Seaweed (g)	9.0		16.6	19.7	27.5	28.0	17.1	18.5	
	Seafood (g)	120.5		82.0	51.0	78.3	41.5	76.9	58.5	
	Meat (g)	166.5		60.9	35.5	57.9	38.5	55.5	36.3	
	Meat and seafood (g)	287.0		142.9	56.5	132.6	56.7	134.5	62.8	
	Eggs (g)	69.5		39.8	36.4	39.3	27.1	39.6	34.1	
Macronutrient intake	Energy (kcal)	2691		1980	383	1796.9	429.8	1635	367	
	Protein (g)	98.9		68.9	15.1	72.7	18.8	63.9	17.7	
	Protein density (%kcal)	14.8		14.0	2.3	16.3	2.0	15.7	2.3	
	Total fat (g)	80.6		50.8	13.7	57.0	23.7	49.8	16.9	
	Fat density (%kcal)	26.9		23.2	5.8	27.7	6.9	27.1	6.3	
	Carbohydrate (g)	380.6		269.5	66.8	244.2	57.5	223.9	51.6	
	Carbohydrate density (%kcal)	58.4		62.7	6.2	56.0	7.4	57.3	7.3	
Micronutrient intake	Potassium (mg)	2701		2622	925	3100	1133	2436	697	*
	Calcium (mg)	899		524	193	663.2	313.0	538.5	260.6	
	Magnesium (mg)	300		270	100	320.9	116.3	242.8	73.2	**
	Phosphorus (mg)	1543		1024	245	1112.3	331.2	964.9	282.1	
	Iron (mg)	9.3		7.6	1.9	9.2	3.3	7.3	2.4	*
	Zinc (mg)	12.4		7.7	1.6	8.3	2.7	7.0	1.9	*
	Copper (mg)	1.5		1.2	0.5	1.2	0.4	1.0	0.3	
	Vitamin A (μgRAE)	623.0		752.6	1414	656.6	314.4	465.9	281.6	
	Vitamin D (μg)	13.4		7.1	5.3	8.5	6.0	8.9	7.3	
	Vitamin E (mg)	7.9		6.9	2.5	7.7	2.3	6.5	2.4	
	Vitamin K (μg)	339.5		221.3	150.0	304.0	189.0	194.6	112.6	*
	Folate (μg)	292.0		353.8	170.9	393.8	123.8	303.2	106.1	**
	Vitamin C (mg)	343.5		107.3	57.3	135.4	55.2	104.7	50.2	
	*n*-3 PUFA (g)	3.1		2.1	1.0	2.3	1.2	2.0	1.1	
	*n*-6 PUFA (g)	10.5		8.6	3.7	9.4	4.4	7.7	3.9	
	Total dietary fiber (g)	13.3		14.5	4.3	17.5	6.3	13.9	4.4	*

*: *p* < 0.05; **: *p* < 0.01: age-adjusted *p*-value; N: number of subjects; SD: standard deviation; SFA: saturated fatty acid; PUFA: polyunsaturated fatty acid.

**Table 2:** Comparisons of BMI, dental parameters, and food and nutrient intake between female dental examination participants eating alone and together.

Category	Health Marker and Foods	Alone (N = 10)		Together (N = 23)		*p*
		Mean, %	SD	Mean, %	SD	
Age and body size	Age (years)	76.5	4.6	73.9	5.9	
	Height (cm)	150.6	5.6	151.5	5.5	
	Weight (kg)	50.9	9.4	49.9	7.9	
	BMI	22.3	3.3	21.7	2.9	
	BMI < 18.5 (%)	10.0		4.3		
	BMI ≥ 25 (%)	20.0		13.0		
Dental status	Present teeth	21.0	7.6	19.9	7.3	
	DMFT Index	16.7	9.4	23.2	5.8	*
	Decayed (untreated)	0.1	0.3	1.0	1.3	*
	Missing teeth	6.8	7.5	8.2	7.3	
	Treated teeth	9.8	6.2	14.0	6.5	
Food intake	Rice (g)	233.9	124.2	199.9	105.4	
	Beans (g)	75.6	69.4	58.1	52.7	
	Green vegetables (g)	157.1	75.4	133.9	83.6	
	Other vegetables (g)	151.2	90.6	137.4	73.0	
	Fresh fruits (g)	151.2	81.6	97.7	62.9	*
	Seaweed (g)	27.5	28.0	17.1	18.5	*
	Seafood (g)	78.3	41.5	76.9	58.5	
	Meat (g)	57.9	38.5	55.5	36.3	
	Meat and seafood (g)	132.6	56.7	134.5	62.8	
	Eggs (g)	39.3	27.1	39.6	34.1	
Macronutrient intake	Energy (kcal)	2011.2	419.0	1623.1	388.0	**
	Protein (g)	82.8	18.6	63.0	17.7	**
	Protein density (%kcal)	16.5	1.6	15.5	2.1	
	Total fat (g)	72.2	20.4	46.5	13.4	***
	Fat density (%kcal)	31.8	5.3	25.6	5.2	**
	Carbohydrate (g)	255.2	57.8	229.4	56.9	
	Carbohydrate density (%kcal)	51.7	5.4	58.9	6.2	**
	Potassium (mg)	3369.6	1075.1	2369.5	629.7	**
	Calcium (mg)	831.8	310.6	507.1	202.4	**
	Magnesium (mg)	351.8	109.1	234.7	64.2	***
	Phosphorus (mg)	1284.4	343.8	946.3	279.9	**
	Iron (mg)	10.9	3.5	6.9	2.1	***
	Zinc (mg)	9.9	2.4	6.8	1.9	***
	Copper (mg)	1.4	0.5	1.0	0.3	*
	Vitamin A (μgRAE)	811.2	322.4	415.0	213.1	**
	Vitamin D (μg)	9.6	5.5	8.4	6.7	
	Vitamin E (mg)	8.4	2.3	6.0	2.4	**
	Vitamin K (μg)	390.5	218.0	183.9	114.0	**
	Folate (μg)	451.5	127.6	294.2	104.1	**
	Vitamin C (mg)	146.6	43.0	104.7	52.7	*
	*n*-3 PUFA (g)	2.8	1.3	1.9	1.0	
	*n*-6 PUFA (g)	11.2	5.1	6.9	2.6	**
	Total dietary fiber (g)	20.0	7.2	13.1	4.1	**

*: *p* < 0.05; **: *p* < 0.01; ***: *p* < 0.001: age-adjusted *p*-value; N: number of subjects; SD: standard deviation; DMFT index: the sum of decayed, missing, and filled teeth; SFA: saturated fatty acid; PUFA: polyunsaturated fatty acid.

**Table 3:** Adjusted increased risks for insufficient dietary intake < lower limit of reference values associated with increasing dental parameters (poorer dental status) in women (N = 33).

Category	Nutrients	Lower Limit	Insufficient (%)	DMFT Index			N of Decayed Teeth			N of Missing Teeth		
				OR	95%CI	*p*	OR	95%CI	*p*	OR	95%CI	*p*
Macronutrient intake	Protein	55 (g)	27.3	1.21	1.00, 1.46	*	2.58	0.96, 6.95		1.08	0.96, 1.22	
	Protein density	14.5 (%kcal)	27.3	1.17	0.98, 1.40		1.54	0.79, 2.97		1.15	1.00, 1.32	
Micronutrient intake	Calcium	500 (mg)	36.4	1.05	0.94, 1.16		1.35	0.72, 2.54		1.07	0.96, 1.20	
	Phosphorus	800 (mg)	24.2	1.04	0.92, 1.17		1.78	0.88, 3.61		1.05	0.93, 1.18	
	Iron	5.5 (mg)	30.3	1.14	0.98, 1.31		1.21	0.65, 2.25		1.02	0.91, 1.14	
	Zinc	6 (mg)	30.3	1.19	1.00, 1.42		1.92	0.88, 4.18		1.09	0.97, 1.22	
	Vitamin A	450 (μgRAE)	51.5	1.07	0.96, 1.18		1.48	0.72, 3.06		1.09	0.96, 1.23	
	Vitamin D	5.5 (μg)	36.4	1.02	0.93, 1.13		1.33	0.71, 2.51		1.06	0.95, 1.19	
	Vitamin E	6.0 (mg)	42.4	1.13	1.00, 1.27		1.60	0.77, 3.30		1.14	0.99, 1.30	
	Vitamin K	150 (μg)	39.4	1.14	1.00, 1.29		1.72	0.81, 3.64		1.09	0.97, 1.23	
	Vitamin B2	1.1 (mg)	27.3	1.12	0.97, 1.30		1.65	0.83, 3.27		1.08	0.96, 1.21	
	Vitamin B12	2.4 (μg)	9.1	1.24	0.80, 1.92		3.45	1.17, 10.1	*	1.13	0.95, 1.35	
	Folate	240 (μg)	24.2	1.07	0.94, 1.21		1.07	0.55, 2.10		1.02	0.90, 1.14	
	Pantothenic acid	5 (mg)	33.3	1.07	0.96, 1.20		1.50	0.77, 2.91		1.09	0.97, 1.22	
	Vitamin C	100 (mg)	36.4	1.04	0.94, 1.16		1.03	0.55, 1.91		1.05	0.94, 1.18	
	*n*-3 PUFA	1.9 (g)	45.5	1.18	1.02, 1.37	*	1.73	0.79, 3.80		1.19	1.01, 1.41	*
	*n*-6 PUFA	7 (g)	48.5	1.16	1.02, 1.32	*	1.64	0.76, 3.54		1.07	0.96, 1.20	
	Total dietary fiber	17 (g)	75.8	1.04	0.94, 1.15		1.04	0.51, 2.14		1.12	0.95, 1.31	
Food intake	Beans	100 (g)	72.7	1.14	1.02, 1.28	*	2.13	0.62, 7.28		1.12	0.96, 1.31	
	Green vegetables	120 (g)	42.4	1.10	0.99, 1.24		1.22	0.65, 2.26		1.17	1.01, 1.36	*
	Other vegetables	230 (g)	87.9	1.05	0.92, 1.19		1.92	0.48, 7.72		1.08	0.89, 1.30	
	Fresh fruits	150 (g)	69.7	1.11	1.00, 1.24		1.59	0.62, 4.07		1.24	1.01, 1.51	*
	Seafood	60 (g)	33.3	1.13	0.98, 1.31		1.77	0.84, 3.71		1.10	0.97, 1.25	
	Meat	50 (g)	42.4	1.05	0.95, 1.16		1.19	0.64, 2.21		1.11	0.98, 1.26	
	Meat and seafood	100 (g)	27.3	1.24	0.99, 1.56		1.66	0.84, 3.31		1.35	1.04, 1.76	*
	Eggs	50 (g)	39.4	0.97	0.88, 1.08		1.32	0.62, 2.82		0.97	0.86, 1.09	

*: *p* < 0.05; age-adjusted *p*-value; N: number of subjects; SD: standard deviation; OR: odds ratio; 95%CI: 95% confidence interval; DMFT index: the sum of decayed, missing, and filled teeth; SFA: saturated fatty acid; PUFA: polyunsaturated fatty acid.

## Data Availability

The datasets used and/or analyzed during the current study are available from the corresponding author on reasonable request.
